# Implantable cardiac pacemaker failure by cumulative dose effects of flattening filter free beams

**DOI:** 10.1093/jrr/rrab041

**Published:** 2021-05-25

**Authors:** Kazuhiko Nakamura, Takahiro Aoyama, Naoki Kaneda, Masashi Otsuji, Yoshitaka Minami, Ami Sakuragi, Masaru Nakamura

**Affiliations:** Department of Radiology, Aichi Medical University Hospital, 1-1 Yazakokarimata, Nagakute, Aichi 480-1195 Japan; Department of Radiation Oncology, Aichi Cancer Center, 1-1 Kanokoden, Chikusa-Ku, Nagoya, Aichi 464-8681 Japan; Graduate School of Medicine, Aichi Medical University, 1-1 Yazako-karimata, Nagakute, Aichi 480-1195 Japan; Department of Radiology, Aichi Medical University Hospital, 1-1 Yazakokarimata, Nagakute, Aichi 480-1195 Japan; Department of Clinical Engineering, Aichi Medical University Hospital, 1-1 Yazakokarimata, Nagakute, Aichi 480-1195 Japan; Department of Radiology, Aichi Medical University Hospital, 1-1 Yazakokarimata, Nagakute, Aichi 480-1195 Japan; Department of Radiology, Aichi Medical University Hospital, 1-1 Yazakokarimata, Nagakute, Aichi 480-1195 Japan; Department of Radiology, Aichi Medical University Hospital, 1-1 Yazakokarimata, Nagakute, Aichi 480-1195 Japan

**Keywords:** FFF beams, implantable cardiac pacemaker (ICP), cumulative dose effects, failure, radiotherapy

## Abstract

Cumulative dose effects, which are one of the main causes of errors that occur when an implantable cardiac pacemaker (ICP) is irradiated with ionizing radiation, induce permanent failure in ICPs. Although flattening filter free (FFF) beams, which are often used in stereotactic radiotherapy, are known to have different characteristics from conventional (with flattening filter [WFF]) beams, the cumulative dose effects on ICPs with FFF beams have been under-investigated. This study investigates ICP failure induced by cumulative dose effects of FFF beams. When the ICP placed in the center of the irradiation field was irradiated with 10 MV-FFF at 24 Gy/min, the cumulative dose at which failure occurred was evaluated on the basis of the failure criteria associated with high cumulative dose as described in the American Association of Physicists in Medicine Task Group 203. The ICP failures such as a mild battery depletion at a cumulative dose of 10 Gy, pacing-output voltage change >25% at a cumulative dose of 122 Gy, and the loss of telemetry capability at cumulative dose 134 Gy were induced by cumulative dose effects. The cumulative doses by which the cumulative dose effects of FFF beams induced ICP failure were not very different from those reported in previous studies with WFF beams. Therefore, radiotherapy with FFF beams (and WFF beams) for patients with ICP requires appropriate management for minimizing the cumulative dose effects.

## INTRODUCTION

Implantable cardiac pacemakers (ICPs) are cardiac implantable electronic devices (CIEDs). ICPs are medical devices implanted in the chest or abdomen that provide electrical stimulation, thereby causing cardiac contraction. An ICP consists of a main unit with a control circuit and battery, which transmits electrical stimulation to the heart. The control circuit consists of a complementary metal oxide semiconductor (CMOS) circuit that consumes less power, thus prolonging the battery life [1]. The control circuit has not only the function of pacing pulse generation and sensing cardiac activity but also a telemetry capability of transmitting and receiving ICP data wirelessly using an external programmer device [2].

Irradiation of CMOS circuits in CIEDs with ionizing radiation can lead to temporary or permanent errors [3,4]. When CIED errors occur, patients may develop shortness of breath, low blood pressure, and in rare cases, serious symptoms requiring cardiopulmonary resuscitation [5,6]. Academic societies have published guidelines for the management of radiotherapy in patients with CIEDs [4,7–10]. One of the latest guidelines, the American Association of Physicists in Medicine (AAPM) Task Group 203 (TG-203), has described the leading cause of CIED errors being dose-rate effects, neutron-induced upsets, and cumulative dose effects [4]. Dose-rate effects can induce transient malfunctions if high dose-rate X-rays are incident on CMOS circuits. Mouton *et al.* reported that 70% of ICPs malfunctioned at dose rates of 8 Gy/min, but no ICPs malfunctioned at dose rates of ≤0.2 Gy/min [11]. The malfunction by dose-rate effects is generally considered as temporary and occurs only during irradiation, hence not inducing permanent failure. However, the malfunctions due to dose-rate effects during irradiation can cause dizziness and fainting in ICP-dependent patients, hence, enhanced patient surveillance is recommended by the AAPM TG-203 [4]. In neutron-induced upsets, the neutron incidence on CMOS circuits inverts the values stored in the memory through a nuclear reaction and can stochastically induce malfunction even at low doses. Grant *et al.* reported that 18 of the 249 patients who received radiation therapy and had implanted CIEDs, all of which used neutron-generating beams, experienced malfunctions [12]. In this report, most of the malfunctions by neutron-induced upsets were transient soft errors that are recoverable through a programmer. Cumulative dose effects can induce permanent failure, such as reduced pacing-output, pacing signal changes, battery drain and loss of telemetry capability owing to the accumulation of abnormal charges caused by repeated irradiation of CMOS circuits. The occurrence of permanent failure not only requires surgical replacement of ICPs but can also cause severe arrhythmias and heart failure in the patient. In previous studies on cumulative dose effects, when ICPs were directly exposed to radiation, ICP failure was observed at cumulative doses ranging from 0.5 to 130 Gy [11,13,14]. The wide range of doses that cause failure indicates that there are the differences in dosage depending on the radiation type, the radiation energy, and the radiation quality [15]. However, the failure that occurs at cumulative dose of ≤1 Gy may be related to neutron-induced disturbances instead of cumulative dose effects [16]. Therefore, it is considered that CIEDs have a higher risk of failure at cumulative doses >5 Gy, and most guidelines recommend cumulative dose thresholds for CIEDs for patients undergoing radiotherapy [4,7–10].

Recently, a linear accelerator that can irradiate flattening filter free (FFF) beams has been developed. An FFF beam increases the dose per pulse by removing the flattening filter, resulting in an increased dose rate. Furthermore, the removal of the flattening filter leads the FFF beam characteristics different from the with flattening filter (WFF) beams, such as increasing the dose rate, softening the X-ray spectrum, increasing the contamination electrons, and reducing secondary neutron production [17]. There are several studies related to CIEDs with FFF beams. Gauter-Fleckenstein *et al.* reported that when CIEDs near the isocenter were irradiated at >100 Gy using volumetric modulated arc therapy (VMAT), no abnormality was observed with 6 MV-FFF, whereas pacing inhibition and data loss were observed with 10 MV-FFF [18]. Nakamura *et al.* reported that all CIEDs directly irradiated with 6 and 10 MV-FFF were induced transient pacing inhibition by dose rates ≥10Gy/min, regardless of the cumulative dose [19]. These studies focus mainly on dose-rate effects and neutron-induced upsets, and it has been reported that there was a difference in the tendency of error occurrence between WFF and FFF beams. However, studies related to cumulative dose effect with FFF beams are limited. Aslian *et al.* performed a partial direct irradiation of CIED with FFF beams using VMAT [20]. No abnormality was observed with 6 MV-FFF in all CIEDs, whereas reprogramming and reduction of pacing pulse width were observed with 10 MV-FFF at cumulative doses of <50 Gy in less than half the CIEDs. Although cumulative dose effects may differ between FFF and WFF due to differences in radiation quality, previous studies have not revealed a relationship between ICP failure due to cumulative dose effect using FFF beams and the doses that induce ICP failure. Since FFF beams can be irradiated at a high dose rate, they are widely used for stereotactic radiotherapy with large doses per fraction [21]. In the future, it is expected that the indication will be expanded to the areas of ventricular tachycardia and spinal metastasis, in addition to lung and liver cancer that have been targeted so far. Therefore, it is useful to report on the cumulative dose effects of FFF beams. This study investigates the ICP failure induced by cumulative dose effects when the CMOS circuit was irradiated with FFF beams.

## MATERIALS AND METHODS

### Irradiation of the ICP

**Fig. 1.**
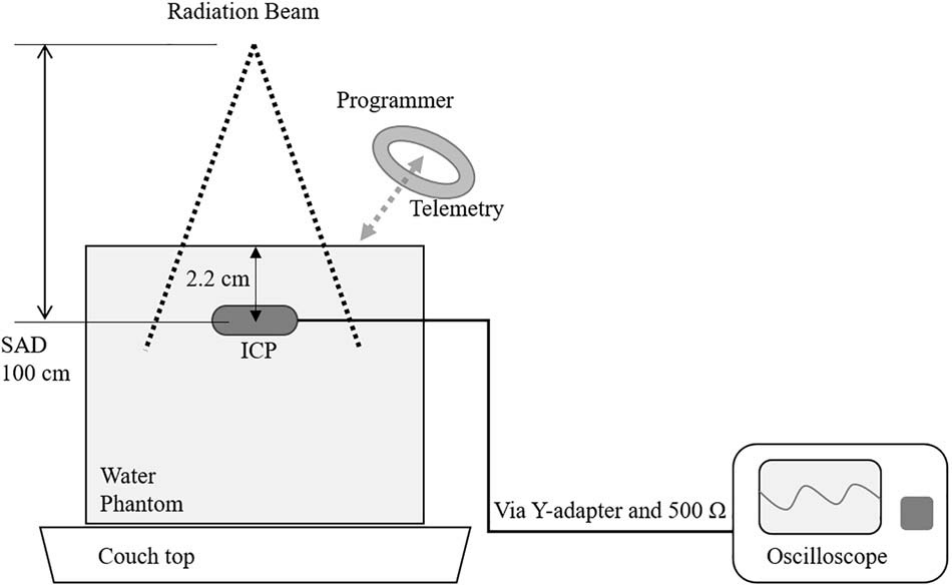
Experimental scheme. The ICP was positioned at a depth of 2.2 cm from the water’s surface in the water phantom filled with water at a source-to-axis distance of 100 cm and at the center of a 10 cm × 10 cm field. ICP parameters were recorded by an oscilloscope via a Y-adapter and a 500 Ω resistance and by telemetry using a programmer. ICP, implantable cardiac pacemaker

An ICP, Sensia SEDR01 (Medtronic, Minneapolis, MN, USA), with no history of use in patients, was used in this study. The MP1 water phantom (PTW, Freiburg, Germany) filled with water was placed on the treatment couch top, and the center of the ICP was placed at a depth of 2.2 cm from the water’s surface. The experimental scheme of the ICP is shown in Fig. 1. TrueBeam STx (Varian Medical Systems, Palo Alto, CA, USA) was calibrated to deliver 1 cGy per 1 monitor unit (MU) at the depth of the maximum dose. When the ICP was installed at the depth of a 2.2 cm maximum dose with 200 MU irradiation using 10 MV-FFF, the absorbed dose of the ICP was defined as 2 Gy in dose to water. The ICP was irradiated with 10 MV-FFF and 24 Gy/min using TrueBeam STx. When the ICP was placed in the center of the 10 cm × 10 cm irradiation field, the ICP was at a source-to-axis distance of 100 cm. Irradiation to the ICP was repeated at a dose of 2 Gy every 1 min and was terminated when ICP parameters could not be sent and received using its telemetry capability.

### Function check of ICP

The ICP was set to VVI mode, which is an ICP setting that senses and paces the ventricle and inhibits pacing-output in response to a ventricular event. The ventricular pulse voltage was set at 5.0 V, rate setting was 60 pulses/min, pulse width was 0.40 ms, with bipolar pacing, and sensing sensitivity of 2.8 mV. The pacing-output voltage of the ICP was measured with an oscilloscope DS-5106B (Iwatsu Electric Co. Ltd., Tokyo, Japan) via a Y-adapter 5866-38 M (Medtronic, Minneapolis, MN, USA) and a resistance of 500 Ω.

ICP parameters were intermittently measured by its telemetry capability using the programmer device Vitatron 2090 (Medtronic, Minneapolis, MN, USA). The measured parameters were battery impedance, battery voltage, lead impedance, pacing pulse width, and pacing signal presence/absence. The battery impedance and battery voltage were used as indexes to evaluate battery depletion. The initial values of each parameter are shown in Table 1.

**Table 1:** Implantable cardiac pacemaker’s parameters and their initial value

Parameter	Value
Battery impedance	375 Ω
Battery voltage	2.69 V
Pacing-output voltage	6.0 V
Lead impedance	514 Ω
Pacing pulse width	0.4 ms

The ICP failure criteria were determined based on the clinical effect associated with high cumulative dose as described in the AAPM TG-203 [4], and failure was judged when any of the following was observed: battery depletion, pacing-output voltage changes >25%, lead impedance changes, loss of telemetry capability, pacing-frequency changes >10% or complete loss of signal.

However, the purpose of this study was to evaluate the dose at which the ICP fails owing to cumulative dose effects; malfunctions that occur temporarily during irradiation were excluded from the evaluation.

## RESULTS

**Fig. 2.**
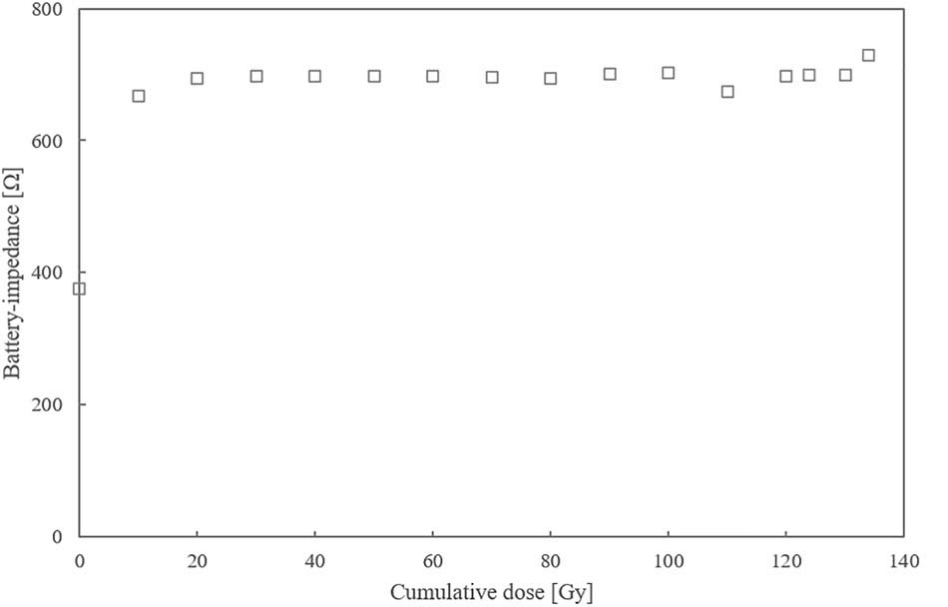
Battery impedance vs cumulative dose. The battery impedance was increased by approximately 77.8% from the initial value of 375 Ω to 667 Ω at a cumulative dose of 10 Gy and changed in the range of −3.1% to 4.7% at a cumulative dose of 10–134 Gy.

**Fig. 3.**
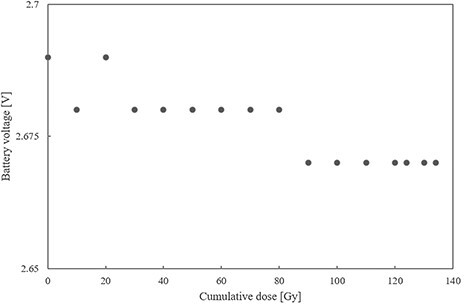
Battery voltage vs cumulative dose. The initial battery voltage was 2.69 V and decreased to 2.68 V and 2.67 V at cumulative doses of 30 and 90 Gy, respectively.

**Fig. 4.**
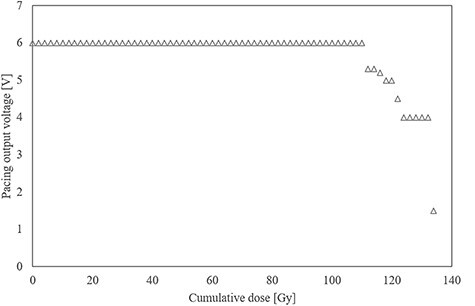
Pacing-output voltage vs cumulative dose. The pacing-output voltage was reduced by >15%, 30%, and 75% from its initial value at cumulative doses of 110, 122, and 134 Gy, respectively.

**Fig. 5.**
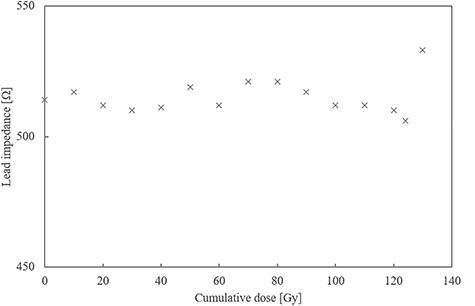
Lead impedance vs cumulative dose. The lead impedance fluctuated in the range of −1% to 4% at a cumulative dose of <130 Gy and became unreadable at a cumulative dose of 134 Gy.

The relationship between each ICP parameter and cumulative dose is shown in Figures 2–5. The battery impedance was increased by about 77.8% from the initial value at a cumulative dose of 10 Gy, and changed in the range of −3.1% to 4.7% at a cumulative dose of 10–134 Gy (Figure 2). The initial battery voltage was 2.69 V, and was decreased to 2.68 V and 2.67 V at cumulative doses of 30 Gy and 90 Gy, respectively (Figure 3). The pacing-output voltage was reduced by >25% from the initial value at a cumulative dose of 122 Gy (Figure 4). The lead impedance became unreadable at a cumulative dose of 134 Gy (Figure 5). At a cumulative dose of 134 Gy, no change from the initial value was observed in the pacing pulse width and pacing signal. Some ICP parameters could not be transmitted and received by the telemetric capability at a cumulative dose of 134 Gy.

## DISCUSSION

This study revealed the dose at which the ICP fails owing to cumulative dose effects by investigating the relationship between the cumulative dose and each ICP parameter when the CMOS circuit was directly irradiated with FFF beams. To the best of our knowledge, this is the first study focused on the cumulative dose effects of FFF beams.

First, the failure criterion was met at a cumulative dose of 10 Gy, and the battery impedance increased about 1.8 times the initial value, indicating battery depletion (Figure 2). In a previous study with WFF beams at a cumulative dose of 10 Gy, an ICP that had sufficient battery capacity before the start of the experiment had an unexpected battery drainage [22]. In our study, battery depletion might have occurred by setting the pacing-output as high, giving priority to the observation of the pacing-output voltage with an oscilloscope. Mouton *et al.* reports that an approximately double increase in battery impedance will not disturb the ICP operation [11]. In this study, we observed only a minor decrease in the battery voltage, which had little to no effect on the output of the ICP. Furthermore, mild battery depletion has a minimal effect on the patients’ quality of life. However, battery depletion could reportedly be observed one week after irradiation [13]. Therefore, had the observation period after irradiation been extended in our study, an ICP failure caused by further battery drain might have occurred and would have required surgical replacement of the ICP.

The failure criterion was met at a cumulative dose of 122 Gy, and the pacing-output voltage was reduced by >25% from the initial value (Figure 3), after which the pacing-output voltage could not be returned to its initial value. It is considered that the permanent failure was induced by the deterioration of the pacing pulse generation function owing to cumulative dose effects caused by repeated irradiation to the CMOS circuit of the ICP. The reduced output voltage is a major failure that can have fatal effects on patients with arrhythmia due to pacing failure. Moreover, this finding was similar to the findings reported in previous studies using WFF beams in which the output voltage of an ICP was reduced when exposed to cumulative doses of >120 Gy [11,13].

At a cumulative dose of 134 Gy, the lead impedance became unreadable by the programmer (Figure 4). This phenomenon was probably because some of the telemetry capabilities were damaged by cumulative dose effects. A previous study has also reported the loss of telemetry capability that occurred at a cumulative dose >120 Gy with WFF beams [13].

In our study, ICP directly irradiated with FFF beams showed signs of failure at a cumulative dose of 10, 122 and 134 Gy, and severe failures were observed at a cumulative dose of 122 Gy. These results were similar to that reported in previous studies with WFF beams [11,13,14]. Moreover, the results were also consistent with that reported by Hurkman *et al.* in which severe failures occurred in the ICP, such as pulse-amplitude changes >25% at cumulative doses of 120 Gy and the loss of telemetry capability at cumulative doses of 130 Gy [13]. However, in a similar study with 10 MV-FFF, CIEDs included in a portion of the irradiated field were observed to have pacing rate reprogramming, pacing threshold reprogramming, and pulse width reduction, at a cumulative dose of 26, 42.7 and 46.4 Gy, respectively [20]. It was different from ICP failure and the cumulative dose at which ICP failure was induced, compared to our study. Their experimental method may have made a difference in our study because the beam incident direction was not unidirectional due to VMAT, and the irradiated volume contained only a part of the CIED’s main unit. Therefore, during instances of the same experimental method, it is considered that there is not a large difference between FFF beams and WFF beams in terms of cumulative doses that induces ICP failure owing to cumulative dose effects.

ICPs have been used as a non-drug therapy in patients with arrhythmias and the number of patients is expected to increase significantly by 2023 [23]. In the future, the number of patients with an implanted ICP requiring radiotherapy may increase. Furthermore, since the number of cases of stereotactic body radiotherapy is increasing each year, revision of the guidelines for radiotherapy patients with ICPs and defibrillators considering the cumulative dose effects of FFF beams is needed.

There are two limitations in this study. First, this study was validated using only a single device. We evaluated the exact cumulative dose effect of the ICP that was not exposed to diagnostic levels of radiation by using a device that had never been implanted in patients. The ICP model verified in this study, although not the latest generation, is currently widely used in clinical practice and was able to provide useful information. The sensing algorithm and ferromagnetic material differ slightly depending on the ICP model and manufacturer, but the basic structure of the CIED does not vary. In future, additional experiments incorporating multiple devices from various models and manufacturers should be undertaken to provide more detailed information. Second, the effects of complex interactions other than the cumulative dose effect have not been completely ruled out. The neutron fluence produced by a 10 MV-FFF beam is approximately 37% lower than that of a 10 MV-WFF beam, although it was difficult to completely separate the effect of neutrons on CMOS circuits from the cumulative dose effect [24]. In addition, the effects of dose rates are considered temporary, although this may not always be the case [4]. Therefore, it is undeniable that neutron-induced upsets and dose-rate effects may have contributed to the failure that we see in our study.

In this report, the doses that induced ICP failure by the cumulative dose effect owing to direct irradiation with FFF beams were 10, 122 and 134 Gy based on the failure criteria associated with a high cumulative dose as described in the AAPM TG-203. The failures were mild battery depletion (with 10 Gy), reduced pacing-output voltage (with 122 Gy) and the loss of telemetry capability (with 134 Gy). Although the characteristics of FFF beams are different from those of WFF beams, the doses at which the ICP failure was induced were comparable to those reported in previous studies with WFF beams. Therefore, radiotherapy with FFF beams (and WFF beams) for patients with an implanted ICP requires appropriate management for minimizing the cumulative dose effects.

## CONFLICT OF INTEREST

The authors declare they have no conflicts of interest.
